# Median Nerve Palsy Caused by a Brachial Artery Pseudoaneurysm Following an Acute Penetrating Trauma

**DOI:** 10.7759/cureus.69254

**Published:** 2024-09-12

**Authors:** Naasik A Muhammad, Abdul Samad, Maria Abdul Rehman, Shazia Yusuf, Zainab Abdul Rahman

**Affiliations:** 1 Emergency Department, Evercare Hospital Lahore, Lahore, PAK; 2 Medicine, Riphah International University, Islamabad, PAK; 3 Diagnostic Radiology, Capital Hospital, Islamabad, PAK; 4 Medicine, Quaid-e-Azam Medical College, Bahawalpur, PAK

**Keywords:** brachial artery pseudoaneurysm, ct angiography, doppler ultrasound, median nerve palsy, trauma

## Abstract

Arterial pseudoaneurysms are uncommon vascular lesions resulting from a breach in the arterial wall leading to contained haematoma formation, often associated with trauma or iatrogenic procedures. Brachial artery pseudoaneurysms following acute penetrating trauma are rare, with even fewer cases presenting with associated median nerve complications. We present the case of a 41-year-old man who developed median nerve palsy secondary to a brachial artery pseudoaneurysm following a knife stab wound to his left cubital fossa. Initially, the pseudoaneurysm was undiagnosed, and the patient experienced worsening symptoms, leading to further investigation and subsequent surgical repair of the pseudoaneurysm. This case underscores the importance of considering vascular injuries and their delayed sequelae in cases of penetrating trauma, particularly when associated with neurological deficits. Prompt diagnosis and intervention are crucial to prevent potential complications and optimise patient outcomes. The utilisation of appropriate imaging modalities, such as Doppler ultrasonography and CT angiography, facilitates accurate diagnosis and guides tailored management strategies. Further research is warranted to explore optimal treatment approaches and long-term outcomes in similar cases.

## Introduction

An arterial pseudoaneurysm is the result of a breach in the arterial wall and the formation of a contained hematoma, either in the adjacent soft tissue or between the tunica media and tunica adventitia layers of the vessel [1]. Unlike true aneurysms, which are caused by an abnormal dilation of all three layers of the arterial wall, pseudoaneurysms are typically characterised by the formation of a sac within the hematoma, with its walls being formed by a mesh of platelet and fibrin crosslinks [2]. The lumens of the pseudoaneurysm and the artery communicate with each other via a neck resulting in turbulent blood flow within the sac [3].

Pseudoaneurysms of peripheral arteries are generally a rare delayed sequela of chronic repeated trauma to the vessel, often secondary to chronic intravenous (IV) drug abuse. They may also be caused iatrogenically, being the second most common complication of procedures requiring an arterial puncture [4]. Other causes include systemic diseases such as giant cell arteritis and Behcet’s disease, fractures, crutch use, and blunt trauma [5]. While pseudoaneurysms can be asymptomatic and eventually spontaneously thrombose and resolve, they may also increase in size, bleed, rupture, exert mass effects on adjacent structures, cause distal embolisation, or become infected, if left untreated, resulting in significant morbidity and mortality [3,6].

Treatment options include pressure bandages applied over the pseudoaneurysm, ultrasound-guided compression of the pseudoaneurysm, ultrasound-guided thrombin injection (UGTI), and open surgical repair [6].

We present the case of a 41-year-old man who developed median nerve palsy secondary to the formation of a brachial artery pseudoaneurysm following an acute penetrating injury. Whilst brachial artery pseudoaneurysms are not entirely uncommon in the literature, the majority of reported cases have been caused by chronic microtrauma, humeral fractures, or iatrogenic. Cases reported to be caused by a single acute penetrating trauma are rare, and those with associated median nerve complications are even more so.

## Case presentation

A 41-year-old man with an unremarkable medical history presented to a local district hospital in Bagh, Kashmir, Pakistan, an hour after sustaining a knife stab wound to his left cubital fossa during an altercation. The patient’s elbow had been tightly wrapped with strips of material to stem the bleeding. At presentation, the bleeding had stopped and the patient was haemodynamically stable with a palpable left radial pulse. After the initial inspection and cleaning of the wound, the laceration was sutured and the patient was discharged.

The following day, the man developed swelling, ecchymosis, tenderness, and severe pain along his entire left forearm and especially in his left hand. He visited his local physician and was prescribed analgesics and non-steroidal anti-inflammatory drugs (NSAIDs). These, however, failed to relieve his symptoms, and one week later he decided to seek care in Islamabad. He presented to the Capital Hospital in Islamabad and underwent a radiograph of his left elbow, which failed to reveal any further anomalies. His wound was inspected and determined to be healing satisfactorily, and he was sent home with additional analgesic medication.

Several days later he presented to a private surgical clinic with unrelenting pain, paraesthesia, hypoesthesia, and marked weakness in his left hand, particularly in the lateral three digits. An electrophysiological study was performed which showed significantly reduced motor and sensory amplitudes, latencies, and conduction velocities in the left median nerve, along with evidence of mild axonal injury at the level of the elbow. He was diagnosed with left median nerve palsy. Subsequently, he was admitted, and his left median nerve was surgically released, both at the elbow and at the carpal tunnel. Thereafter, he was discharged with analgesic medication and a physiotherapy referral. For the following four months, he underwent physiotherapy and follow-up consultations with the surgeon, and he was assured that, in time, his nerve injury would heal. Despite some reported relief in his symptoms, particularly pain, his hand had not regained much functionality.

Approximately seven months after his initial presentation, the man presented to the Capital Hospital in Islamabad with a painless, palpable, and pulsatile soft tissue swelling on the anteromedial aspect of the left elbow. He reported that the swelling had gradually increased in size over the past few months. He also reported that while he was mostly pain-free, he did experience decreased sensation and significant weakness in his left hand. On examination, he had difficulty in flexing his wrist and lateral three digits. An electrophysiological study was conducted again which showed severe axonal injury to the left median nerve distal to the innervation of the pronator teres and partial axonal injury to the left ulnar nerve at the level of the innervation of the flexor carpi ulnaris.

Ultrasonography was performed, which revealed the following: a large, round, well-circumscribed, thick-walled, heterogeneously hypoechoic lesion in the subcutaneous plane on the anteromedial aspect of the left elbow, measuring 34mm anteroposteriorly x 37mm transversely x 45mm longitudinally; in close proximity to, and communicating with, the left brachial artery via a narrow neck measuring approximately 5mm. Colour Doppler showed bidirectional turbulent flow due to the swirling of blood within the lesion, creating a yin-yang pattern. Spectral Doppler showed a bidirectional to-and-fro pattern of blood flow.

These sonographic findings were suggested to be compatible with a pseudoaneurysm arising from the left brachial artery. While the initial ultrasound was highly suggestive of a pseudoaneurysm, a CT angiogram was performed to obtain more detailed anatomical information, crucial for surgical planning and to better delineate the extent of the vascular injury and its relationship to surrounding structures.

**Figure 1:**
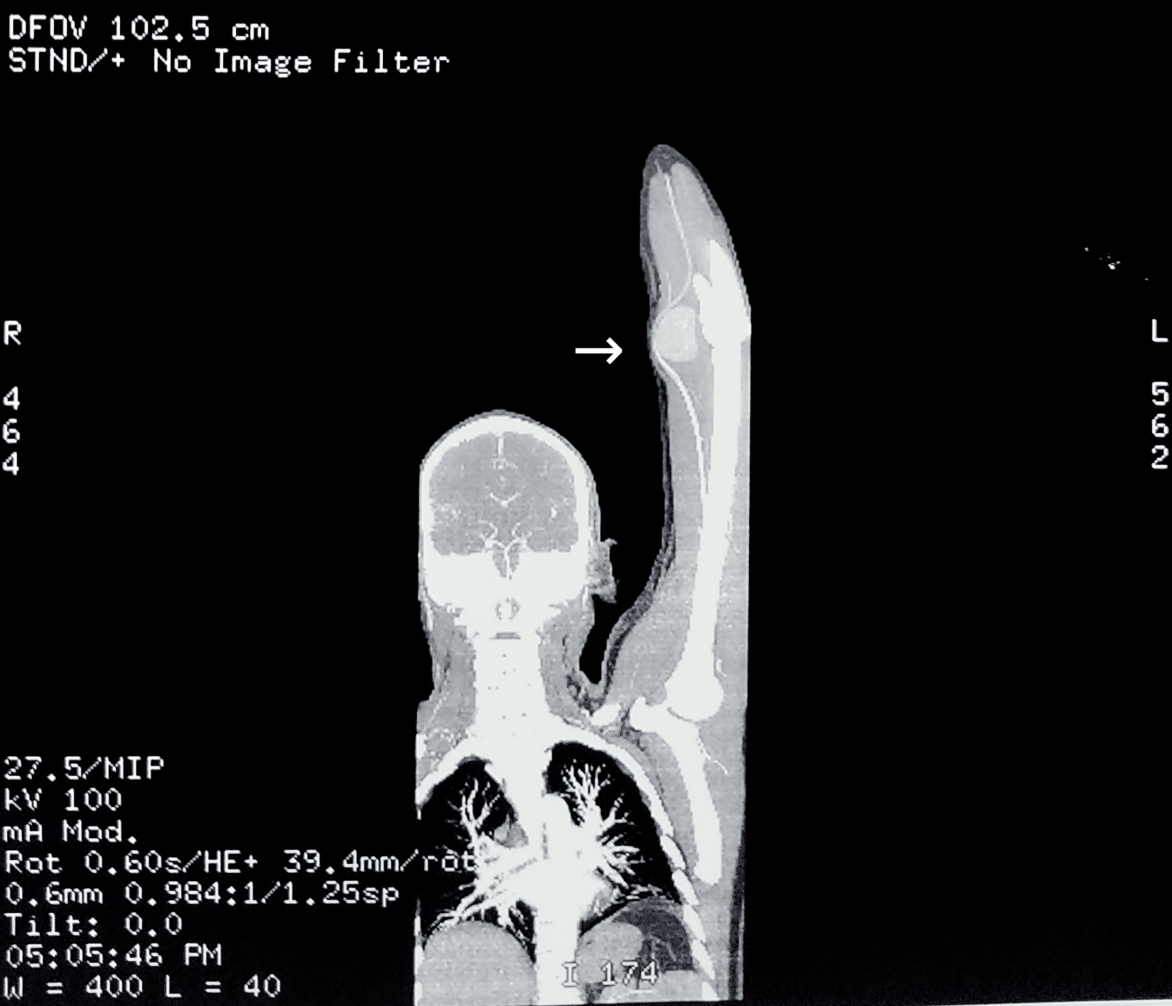
CT angiogram (coronal section) The image shows a well-defined aneurysmal outpouching, with complete contrast opacification, arising from the distal part of the brachial artery.

**Figure 2:**
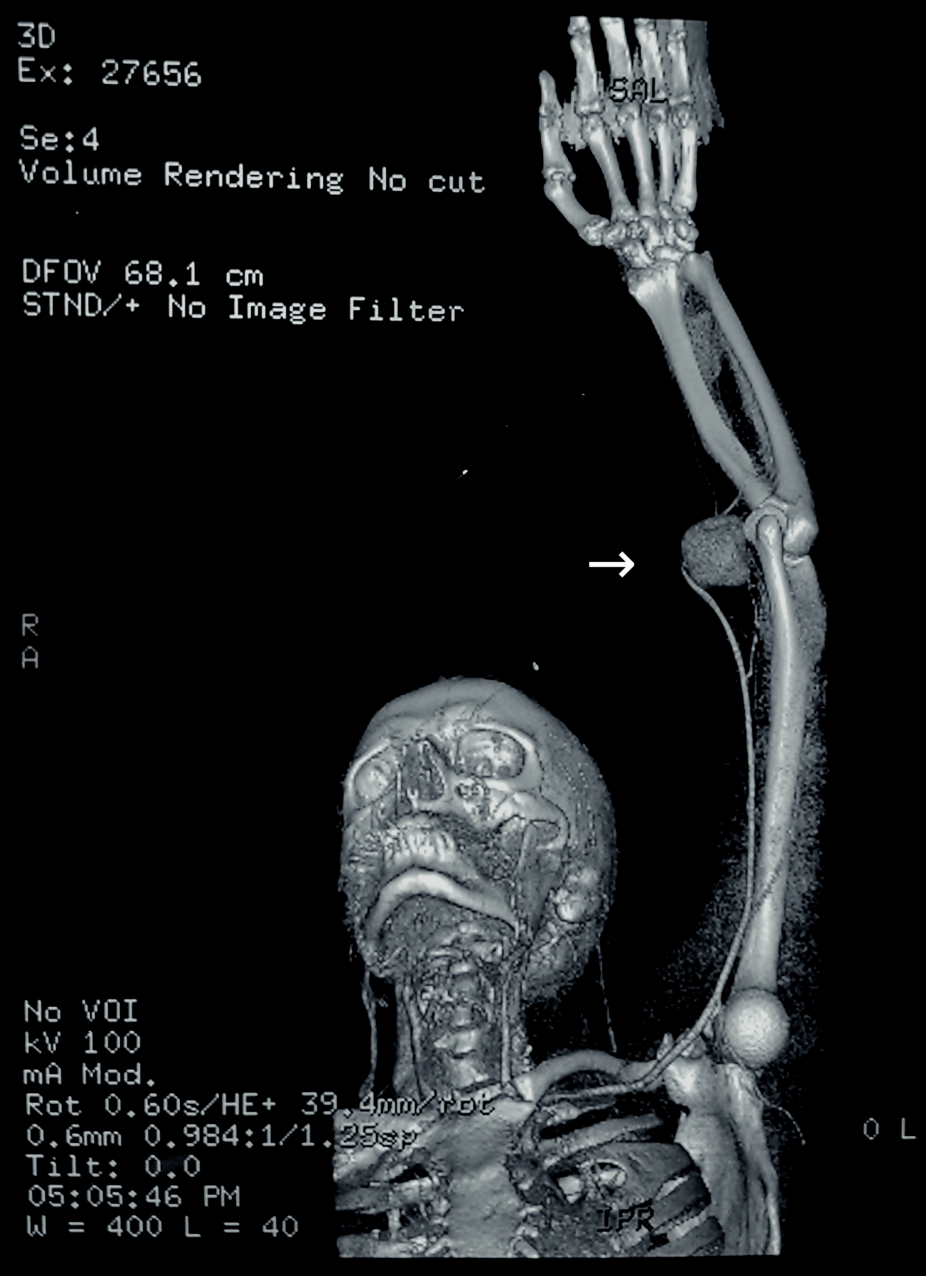
3D reconstructed angiogram The angiogram demonstrates a well-defined pseudoaneurysm arising from the distal part of the left brachial artery, just proximal to its bifurcation in the cubital fossa.

The CT angiography of the left upper limb showed a well-defined aneurysmal outpouching arising from the distal part of the brachial artery, just proximal to its bifurcation in the cubital fossa (See Figures 1,2), measuring 4.2cm x 3.2cm x 4.2cm. It showed thickened, non-enhancing walls suggestive of peripheral thrombosis. The ulnar and radial arteries appeared normal in calibre, with normal contrast opacification. Free flow of contrast was seen in the distal vessels of the hand. A definitive diagnosis of a pseudoaneurysm arising from the left distal brachial artery following acute penetrating trauma was made. The case was then referred to a vascular surgeon for repair of the arterial pseudoaneurysm under general anaesthesia. After the necessary optimisation for the procedure, the pseudoaneurysm was excised and the artery was repaired using an interposed reverse basilic vein graft.

Doppler ultrasonography was repeated one month after the procedure, which showed the venous graft connecting the distal portion of the brachial artery with the proximal portion of the ulnar artery in the medial cubital fossa and proximal forearm, measuring approximately 40mm in length. A reversed valve was noted within the graft. Colour and spectral Doppler analysis showed a laminar and monophasic waveform with a peak systolic velocity of 38cm per second. The patient reported significant improvement in his left-hand function and sensation.

## Discussion

We have discussed the case of a 41-year-old man who developed median nerve palsy secondary to the formation of a brachial artery pseudoaneurysm following an acute penetrating injury. The median nerve was surgically released, albeit without prior ultrasonographic evaluation or through vascular exploration during the procedure. Subsequent to this, although pain relief was achieved, the functional capacity of the hand did not improve. Several months after the initial presentation, the pseudoaneurysm increased in size and became clinically apparent, whereupon it was confirmed sonographically and radiologically. An electrophysiologic study showed not only an increased median nerve deficit but also an ulnar nerve deficit. The patient underwent surgery for the excision of the pseudoaneurysm and reconstruction of the brachial artery using an interposed venous graft.

This case demonstrates one of the rare potential complications of penetrating trauma to the upper limb. Pseudoaneurysms are less common in the upper limb as compared to the lower limb [7], and post-traumatic pseudoaneurysms have an estimated incidence of less than 0.5% [8]. The classical presentation of peripheral artery pseudoaneurysms is a painful, tender, and pulsatile swelling, which should prompt further investigation. However, pseudoaneurysms which result as a sequelae of penetrating trauma, can have a delayed presentation, often presenting months or even years after the initiating trauma [8]. This case is unique in two aspects: firstly, it presented with a painless swelling, unlike the classical presentation of painful, pulsatile masses associated with pseudoaneurysms; secondly, while most cases of median nerve palsy associated with brachial artery pseudoaneurysms are iatrogenic in origin, this case was caused by an acute penetrating injury, distinguishing it from almost all those found in the literature.

Besides peripheral artery pseudoaneurysm, other potential etiologies contributing to similar clinical presentations include hematoma formation, compartment syndrome, abscess formation, vascular malformations, and soft tissue tumours. Hematomas may manifest as localised swelling, but a delayed presentation is unlikely. Compartment syndrome would present with significant distal vascular compromise and elevated compartment pressures. Abscess formation, often secondary to infection, may present as a fluctuant, tender mass accompanied by systemic signs of inflammation. Vascular malformations, such as arteriovenous fistulas or venous aneurysms, can present with pulsatile masses and may require specialised imaging modalities for accurate diagnosis. Lastly, soft tissue tumours can mimic pseudoaneurysms clinically and radiologically, necessitating histopathological evaluation for definitive diagnosis. Careful consideration of the patient's clinical history, physical examination findings, and appropriate diagnostic imaging modalities is crucial in differentiating between these entities to guide tailored management strategies.

The use of Doppler ultrasonography and CT angiography was invaluable in the diagnosis and management of this case. Doppler ultrasonography is currently recognised as the gold-standard imaging modality for the initial detection of peripheral pseudoaneurysms [9].

Ultrasound-guided thrombin injection (UGTI) is currently regarded as the first-line treatment modality, and open surgical repair is reserved for cases that either have not responded to UGTI or in which UGTI is contraindicated [4]. Other treatment options include conservative management, ultrasound-guided compression, and compression bandaging. However in this case open surgical repair was opted for due to the non-availability of resources and the larger size of the pseudoaneurysm.

A critical aspect of this case is the initial failure to diagnose the brachial artery pseudoaneurysm, despite the patient's significant symptoms and history of penetrating trauma. The initial presentation with median nerve palsy should have prompted a thorough investigation for potential vascular injuries, including earlier Doppler ultrasonography or CT angiography. However, these diagnostic modalities were not utilised until the pseudoaneurysm became clinically apparent months later. This delay highlights the importance of maintaining a high index of suspicion for vascular injuries in cases of penetrating trauma, especially when associated with neurological deficits, and the importance of early recognition and prompt management of nerve injuries, which can have significant long-term consequences for patients. The longer the delay in diagnosis, the worse the neurological outcome. Additionally, physicians should be cognizant of the possibility of such a complication developing, even years later. 

## Conclusions

In conclusion, this case highlights the challenges and complexities in diagnosing and managing brachial artery pseudoaneurysms following penetrating trauma. It underscores the importance of maintaining a high index of suspicion for vascular injuries and utilising appropriate diagnostic imaging modalities early in the clinical course. Additionally, it emphasises the need for flexibility in treatment strategies to adapt to individual patient circumstances and resource constraints. Ultimately, prompt recognition and intervention are essential to mitigate potential complications and optimise patient outcomes.
